# The relationship between critical life events, psycho-emotional health and life satisfaction among youths: coping mechanisms and emotional regulation

**DOI:** 10.3389/fpsyg.2023.1288774

**Published:** 2024-01-11

**Authors:** Gabriela Marc, Laurențiu Mitrofan, Camelia-Iulia-Maria Vlad

**Affiliations:** Faculty of Psychology and Educational Sciences, Department of Applied Psychology and Psychotherapy, University of Bucharest, Bucharest, Romania

**Keywords:** coping mechanisms, critical life events, emotional regulation, life satisfaction, psycho-emotional health, youths

## Abstract

**Objective:**

Several studies in the specialized literature have reported that events such as the death of a loved one, job loss, divorce, illness, or retirement lead to an increase in the level of stress felt, and subsequently stress affects the person on several levels of life, such as: personal, relational, social, academic and at the same time sanogenic. The present paper explored the relationship between critical life events and psycho-emotional health among youths and the manner in which this relationship is mediated by the level of life satisfaction.

**Methods:**

The data was extracted using a sample of participants (between 20 and 40 years old) from several cities in Romania, who experienced one or more critical life events during the last year. Data organization and hypothesis testing were performed using IBM SPSS 23 and jamovi programs. For this purpose we used the simple regression analysis, Pearson correlation and mediation analysis. The scales used to conduct the research were: RS-14, SRRS, ERQ, CERQ, DASS-21 and SWLS.

**Results:**

The final sample of the study totaled 190 female and male Romanian participants aged between 20 and 40 (*M* = 24.45, SD = 5.27) who had experienced critical events during the last year, leading to psycho-emotional destabilization and a significantly lower level of life satisfaction. The prevalence of critical life events among these participants varied from one to five events (55.26%) and up to more than 16 critical events in the past year (2.63%). The presence of these events led to increased levels of stress, anxiety, or depression among participants. Furthermore, it appears that the presence of a high number of critical life events led to a decreased life satisfaction among participants, along with a greater tendency toward catastrophizing.

**Conclusion:**

Critical life events are increasingly frequent events in everyday life, and youth seems to be the period with the most changes. The research findings add to current findings about the practical implications that critical life events have on psycho-emotional health among youths. Therefore it appears to be a close relationship between critical life events, psycho-emotional health, and emotional regulation. At the same time, it seems that coping mechanisms have a central role in the level of life satisfaction among youths.

## 1 Introduction

In the scientific community, the focus has been placed over the last years on the study of events that represented a direct threat to the person’s social or economic status, self-esteem or physical and mental well-being. However, in recent years, there has been a gap in the specialized literature not highlighting the fact that critical life events are events that occur more and more often among youths, and they often have a negative effect on their psycho-emotional health. Therefore, the objective of this research is to understand the manner in which critical life events represent a predisposing factor for the destabilization of mental and emotional health among youths. Life has a way of throwing us curveballs, and some of these events can have a profound impact on our lives. When these events bring about high levels of stress or adversity, they can even spill over into our mental health, causing negative effects (Dohrenwend, 2006; Lucas, 2007; Monroe et al., 2007). In the world of research, these events are known as critical life events, as they are seen as threats to our well-being and can harm our psycho-emotional health (Cohen et al., 2019). It is known that any event, regardless of its emotional or affective resonance, causes an imbalance on various levels of life at some point. Therefore, going through significant life events and adjusting to the changes they bring can have immediate impact on our emotions and physical well-being. These events can also influence our long-term psychological well-being, life satisfaction, and our emotional and social functioning (Schwarzer and Luszczynska, 2012).

It is stated that each critical event has its own physical, social or biological requirements to which the human individual must adapt, a process called stress response in the specialized literature (Schwarzer and Luszczynska, 2012). However, individual differences may intervene around the people’s level of perception, when they are exposed to the same event,. As a result, individuals may experience the intensity of the same event differently, leading to potential variations in psycho-emotional consequences among people. Exploring the detrimental impact of critical life events on quality of life is a highly significant subject, particularly considering the findings of numerous studies in the current literature that emphasize this research area. These studies highlight how events categorized as critical can potentially lead to health issues under specific conditions. Therefore, identifying the factors that alter the psychological and emotional balance, and how they can subsequently lead to clinical symptomatology in the sphere of depressive, anxious or traumatic disorders, is a central point of this research. Moreover, the changes in the way of following the individuals’ confrontation with critical life events can affect the level of life satisfaction, defined in the specialized literature as the extent to which a person finds life to be rich, qualitative and full of meaning (APA Dictionary of Psychology, 2022b).

Events that we anticipate will happen at some point in life are: school transitions, moving home, marriage, pregnancy or the death of parents. However, there are also certain events that we rarely come into contact with, but which lead to repercussions in the psycho-emotional sphere if they are perceived as strongly destabilizing. These events include divorce, the loss of a life partner through death, unemployment, legal troubles such as arrest, or even receiving a diagnosis of an incurable disease (Schwarzer and Luszczynska, 2012). They frequently leave profound emotional imprints on individuals, diminishing their overall life satisfaction, and serving as sources of negative implications for their psycho-emotional well-being (Cohen et al., 2019). For this reason, such events are described by the Dictionary of the American Psychological Association as critical life events, which require optimal adaptive behavior so that the psychological and social functioning of any person is not destabilized (APA Dictionary of Psychology, 2022a). Health in not solely determined by physical well-being, but also encompasses a person’s psychological, emotional, and coping aspects in response to life events (Juškelienė et al., 2013). However, the risk of disrupting one’s psycho-emotional balance and potentially developing health issues arises when an event fulfills certain criteria to become a threat to personal well-being or life satisfaction (Cohen et al., 2019). In this context, Dohrenwend’s 2000 study proposed six general characteristics of critical life events that contribute to the nature and extent of changes experienced by individuals affected by these events. These characteristics include whether the events have a positive or negative impact, their source, the degree of unpredictability, their centrality in a person’s life, their magnitude, and their potential to physically or emotionally weaken the individual (Monroe et al., 2007). Furthermore, repeated exposure to stressors is considered the most harmful form of exposure to factors that can impact human health. This is because critical events are those that lead to lasting changes in emotional, and physiological responses, and behavior, ultimately influencing the progression of health conditions (Cohen et al., 2019).

In recent years, clinical research has focused on understanding how stressful life conditions can be an important cause of mental disorders (Schwarz, 2018). However, personality factors and coping mechanisms are the elements that can most significantly favor the felt impact of critical life events. For example, it seems that people whose personality is characterized by a higher level of neuroticism and a lower level of conscientiousness and agreeableness, are more likely to experience a divorce. At the same time, a catastrophizing thinking pattern and avoidant coping mechanisms can cause individuals to experience a greater number of critical life events (Cohen et al., 2019), when the level of personal resilience is diminished.

Psycho-emotional health, as defined in specialized literature, is a subjective construct that indirectly mirrors key aspects of an individual’s personality, including motivations, values, cognitive processes, emotions, and adaptability. It is important to note that psycho-emotional health is not static; it evolves and adapts depending on an individual’s age and the life experiences they encounter. It can be seen as a crucial factor in reducing a person’s psychological vulnerability in situations of risk (Kichula et al., 2023). As the specialized literature shows, major changes in the individual’s life (for example, job loss, the death of a loved one) are associated with increased predisposition to depressive symptoms (Panchal et al., 2021), and major depression is one of the most frequently diagnosed psychological conditions (Monroe et al., 2007) caused by adverse life events. It appears that people who show clinical signs of depression are 2.5–9.4 times more likely to have experienced, before their first depressive episode, a critical life event with an impact that produced a significant level of stress. At the same time, the major changes in the individual’s life resulting from the confrontation with the critical events lead to an increase in the level of anxiety and stress and even to the risk of relapse in the case of major depressive disorder (Cohen et al., 2019) along with a lower level of satisfaction with life. Currently, life satisfaction is also studied as a mediating variable in reducing the level of stress associated with critical life events and coping mechanisms (Friborg et al., 2006; Schaan and Vögele, 2016).

## 2 Methodology

### 2.1 Design

The study design is non-experimental, confirmatory and correlational.

### 2.2 Participants

The initial sample consisted of 204 participants, but the final sample comprised a number of 190 people aged between 20 and 40 who participated in the present study, *M* = 24.45, AS = 5.27. Among them, 171 people are female (90%) and 19 are male (10%). The majority of them come from urban areas (72.6%) and 52 participants come from rural areas (27.4%).

### 2.3 Procedure

The 204 participants in this study were recruited online through the “snowball” method. The form with the study questionnaires was distributed on Facebook with the request to be forwarded. Regarding the distribution of the questionnaire, the purpose of the study was specified in the form, and information regarding the confidentiality of the data was provided to the participants. This is used exclusively for academic purposes.

Inclusion criteria for the study were that the participants were between the ages of 20 and 40 and had experienced critical life events such as the loss of a loved one, divorce, or lifestyle changes during the past year. Of the 204 responses initially received, 14 did not meet the conditions of correctness and completeness because they were either not between the ages of 20 and 40 or did not face critical life events during the last year. These were the reasons why they were excluded from the study, maintaining a number of 190 responses which was considered sufficient for this stage of the research.

The participants of the present study received through the Facebook social network - the form that included a section for the presentation of the study, followed by a section dedicated to the informed consent to which they expressed their agreement to participate in the study. The following sections of the form included questions on sociodemographic data and a set of six questionnaires intended for completion. After going through the sections related to the study, the participants benefited from the possibility of participating in a raffle with a shopping voucher as a prize. Following the completion of the entire sequence of steps in this study, participants submitted the form with the answers to the research questionnaires, completing the data collection step. Participants were asked to complete the informed consent form expressing their agreement to participate in the study. The data contained in this study is part of a dissertation thesis, which has undergone a careful analysis, meeting the necessary ethical criteria.

### 2.4 Instruments

The representative sociodemographic variables for this study, were: age, gender, background and level of completed studies.

The number of critical life events was measured with the Social Readjustment Rating Scale (SRRS) (Holmes and Rahe, 1967). The Social Readjustment Rating Scale (SRRS) was originally developed to investigate how life events leading to a significant level of stress may lead to a susceptibility among people to poor mental health in the future. The SRRS scoring system includes 43 life events, each assigned a score from 0 to 100 units of life change (ULC), with each participant having to tick each type of event on which was exposed during the last year. Subsequently, two global scores are obtained, represented by the life events index that outlines the total number of events experienced by the person and the social readjustment index obtained by adding all life change units (Blasco-Fontecilla et al., 2012). According to the authors Holmes and Rahe (1967), if the score obtained is in the range of 0–149 ULC, there are no significant problems in the life of the person concerned. If the score varies between 150 and 300 ULC there is a 50% chance that the person in question will have problems in the psycho-emotional sphere in the next 2 years, the risk being even higher (80%) if the total score exceeds 300 ULC. The scale was translated to Romanian, and the items were formulated in such a way as to capture critical life events to which the participants were exposed during the last year. For example, an item included: “Death of spouse (100 ULC).” In our study, the internal consistency analysis obtained a Cronbach Alpha coefficient *α* = 0.88.

Emotional regulation capacity was measured with the Emotion Regulation Questionnaire (ERQ, Gross and John, 2003). The questionnaire totals 10 items, which are grouped into two dimensions: Reappraisal (items 1, 3, 5, 7, 8, 10) and Suppression (items 2, 4, 6, 9). The questionnaire items are scored on a 7-point Likert scale, where 1 = strongly disagree, and 7 = strongly agree. Sample items: “When I want to have more positive emotions (such as joy or amusement), I change the thing I was thinking about.” The questionnaire was translated and adapted for the Romanian population by Heilman (2014). In our study, the internal consistency analysis obtained a Cronbach Alpha coefficient *α* = 0.89 for the reappraisal subscale and α = 0.86 for the suppression subscale.

Cognitive-emotional coping mechanisms were measured with the Cognitive Emotion Regulation Questionnaire (CERQ) (Garnefski and Kraaij, 2007). The questionnaire totals 36 items, which are grouped into 9 subscales - Self-blame (items 1, 10, 19, 28), Acceptance (items 2, 11, 20, 29), Rumination (items 3, 12, 21, 30), Positive refocus (items 4, 13, 22, 31), Refocus on planning (items 5, 14, 23, 32), Positive reappraisal (items 6, 15, 24, 33), Putting into perspective (items 7, 16, 25, 34), Catastrophizing (items 8, 17, 26, 35), Blaming others (items 9, 18, 27, 36). Items are scored on a 5-point Likert scale, where 1 = almost never and 5 = almost always. Sample item: “I often think about how I feel about what happened to me.” The questionnaire was translated and adapted for the Romanian population in the study conducted by Heilman (2014). In our study, internal consistency coefficients ranged between *α* = 0.88 and *α* = 0.92 for the instrument’s nine subscales.

Psycho-emotional health was measured with the Depression Anxiety Stress Scales (DASS-21) (Lovibond and Lovibond, 1995). The scale totals 21 items grouped into three subscales (Depression– items 3, 5, 10, 13, 16, 17, 21, Anxiety– items 2, 4, 7, 9, 15, 19, 20 and Stress– items 1, 6, 8, 11, 12, 14, 18). Items are scored on a 4-point Likert scale, where 0 = did not happen to me at all and 3 = happened to me a lot or most of the time. Example item: “It was hard for me to calm down.” The instrument was translated into Romanian, and the items were formulated in such a way as to capture the emotional state of the participants in the last week. In our study, the internal consistency analysis obtained high coefficients, Cronbach Alpha for the Stress subscale being *α* = 0.90, *α* = 0.88 for the Anxiety subscale and *α* = 0.94 for the Depression subscale.

Participants’ level of satisfaction with their own lives was measured with the Satisfaction With Life Scale (SWLS) (Diener et al., 1985). The scale consists of 5 items scored on a 7-point Likert scale, where 1 = Strongly Disagree and 7 = Strongly Agree. Example item: “In general, my life is close to my ideal.” In the present study, when analyzing the internal consistency, a Cronbach Alpha coefficient *α* = 0.91 was obtained.

### 2.5 Analysis of data

The data collected through the questionnaire were subsequently subjected to statistical analysis. Data organization and hypothesis testing were performed using IBM SPSS 23 (IBM Corp, 2015) and jamovi (The jamovi project, 2023). In this regard, the simple regression analysis, Pearson correlation and mediation analysis were used. The results obtained in the research highlighted the fact that there is a close relationship between critical life events, psycho-emotional health and life satisfaction among youths.

## 3 Results

### 3.1 Sample characteristics

Among the 190 participants of sample, 91 have high school education (47.9%), 80 have bachelor’s education (42.1%), 16 have master’s education (8.4%) and three people have doctoral education (1.6%). In terms of occupation, 129 participants are students (67.9%), 55 participants are employed (28.9%), three participants are neither currently studying nor employed (1.6%), and another three participants are looking for a job (1.6%).

With regard to the number of critical life events that the participants faced in the past year, 105 participants (55.26%) faced one to five events, 80 of the participants (42.11%) stated that they faced with six to 15 critical events, and 5 of the participants (2.63%) stated that they had experienced more than 16 critical events in the past year. The most frequently mentioned events were related to preparation for certain planned events such as vacations or important holidays, followed by major changes in sleep schedule and eating habits, as well as moving home and revising personal habits regarding changes in clothing style or giving up drinking of tobacco.

Regarding the impact of the estimated critical events to which the participants were exposed during the last year, 120 of them (63.16%) declared that they were exposed to less critical events totaling between 11 and 150 life-changing units (units of life change - ULC) which translates into low chances of having their psycho-emotional health and life satisfaction affected in the near future. A total of 52 participants (27.36%) stated that they had been exposed to critical events totaling between 151 and 300 life-changing units, and, according to Holmes and Rahe (1967), these participants have more than a 50% chance of showed poor psycho-emotional health in the next 2 years. At the same time, following the findings, it seems that 18 of the participants (9.47%) were exposed to multiple critical events, totaling more than 300 life-changing units, which indicates that they have more than 80% chance of facing poor psycho-emotional health in the next 2 years.

### 3.2 Descriptive statistical analysis

The mean scores, standard deviations, internal consistency coefficients, indices of symmetry and flattening among the analyzed variables are presented in Table 1.

**Table 1:** Mean scores, standard deviations, internal consistency coefficients, indices of symmetry and flattening among the analyzed variables.

Variables	*M*	SD	*α*	Skewness	Kurtosis
1. Critical life events	142.97	100.99	0.88	1.24	1.50
2. Emotional regulation-reevaluation	5.12	1.14	0.89	−0.27	−0.59
3. Emotional regulation-suppression	3.66	1.57	0.86	0.07	−0.98
4. Self-blame	11.99	4.12	0.88	0.24	−0.66
5. Acceptance	15.17	3.67	0.83	−0.41	−0.78
6. Rumination	15.61	3.58	0.88	−0.72	0.32
7. Positive refocusing	13.22	3.94	0.90	−0.05	−0.60
8. Refocus on planning	16.41	2.80	0.82	−0.53	−0.16
9. Positive review	15.78	4.03	0.92	−0.81	−0.09
10. Perspective	15.20	4.22	0.91	−0.72	−0.23
11. Catastrophizing	9.86	4.56	0.89	0.39	−0.85
12. Blaming others	9.54	3.83	0.90	0.41	−0.22
13. Stress	11.65	5.41	0.90	−0.01	−0.83
14. Anxiety	8.63	5.74	0.88	0.18	−0.91
15. Depression	8.21	6.31	0.94	0.28	−1.11
16. Life satisfaction	24.18	6.46	0.91	−0.33	−0.27

### 3.3 Inferential statistics – hypothesis testing

The outcomes of the regression analysis unveiled a noteworthy positive association between the absence of critical life events and psycho-emotional well-being, specifically in terms of stress levels among young individuals. Remarkably, the lack of critical life events accounted for 7% of the variance in stress levels, which serves as a representative measure for psycho-emotional well-being [*R*^2^ = 0.07, *F* (1, 188) = 14.07, *p* < 0.01, *β* = 0.26]. The corresponding confidence interval for this relationship was calculated to be 95% CI [0.01, 0.02], as detailed in Table 2, Model 1. Regarding the connection between the absence of critical life events and anxiety, the regression analysis outcomes demonstrated a positive linkage. Specifically, the absence of critical life events accounted for 3% of the variance in anxiety levels, a representative metric for psycho-emotional well-being [*R*^2^ = 0.03, *F* (1, 188) = 5.74, *p* > 0.01, *β* = 0.17]. The confidence interval for this association was calculated to be 95% CI [0.00, 0.02], as presented in Table 2, Model 2.

**Table 2:** Regression coefficient for critical life events as a predictor of psycho-emotional health in terms of stress, anxiety and depression.

Model	*R* ^2^	*B*	ES B	*β*	*t*	*p*
1	0.07	0.01	0.00	0.26	3.71	0.00
2	0.03	0.01	0.00	0.17	2.40	0.02
3	0.44	0.01	0.00	0.21	2.40	0.00

1 – stress, 2 – anxiety, 3 – depression.

Regarding the relationship between the absence of critical life events and depression, the results of the regression analysis showed that the absence of critical life events is positively associated with psycho-emotional health in terms of the level of depression among youths. Thus, the absence of critical life events explains 44% of the level of depression as a representative variable for psycho-emotional health, *R*^2^ = 0.44, *F* (1, 188) = 8.62, *p* < 0.01, *β* = 0.21, the confidence interval being 95% CI [0.00, 0.02] (Table 2, Model 3). The absence of critical life events is a significant positive predictor of psycho-emotional health among youths, which indicates that the hypothesis is confirmed.

The results of the statistical analysis show that we obtained a significant negative correlation between coping mechanisms focused on catastrophizing and resilience (*r* = −0.22; *p* < 0.05). This result translates into the fact that a greater tendency towards catastrophizing is associated with a lower level of life satisfaction and vice versa (Table 3).

**Table 3:** Pearson correlations for catastrophizing coping mechanisms and life satisfaction.

	Catastrophizing	Life satisfaction
Catastrophizing	1	
Life satisfacțion	−0.217**	1

*provided in semnificatives coefficients for α<0.01.

Starting from the results obtained following the statistical analysis regarding emotional regulation, it is observed that p < 0.01 for stress (*r* = −0.28) and depression (*r* = −0.31), and for anxiety (*r* = −0.22) *p* > 0.01, which translates into the existence of a statistically significant negative correlation between emotional regulation in terms of reappraisal and psycho-emotional health. We can thus state that a low level of stress, anxiety and depression is associated with a higher degree of reevaluation among youths. Also, following the statistical analysis, it is observed that *p* > 0.01 for stress (*r* = 0.11) and depression (*r* = 0.35) and *p* < 0.1 for anxiety (*r* = 0.13), which translates into the absence of a statistically significant positive correlation between emotional regulation in terms of suppression and psycho-emotional health. We can thus state that a high level of stress, anxiety and depression is not associated with a lower degree of suppression among youths (Table 4).

**Table 4:** Pearson correlations for emotional regulation and psycho-emotional health.

	Stress	Anxiety	Depression	Revaluation	Suppression
Stress	1				
Anxiety	0.64	1			
Depression	0.65	0.63	1		
Revaluation	−0.28	−0.22	−0.31	1	
Suppression	0.11	0.13	0.35	−0.12	1

^*^p < 0.05; ^**^p < 0.01; ^***^p < 0.001.

It was found that life satisfaction, with a mediation percentage of 90.54%, mediates the association between stress and concentration on planning. The estimated amount of mediation is −0.04, 95% confidence interval [−0.07, −0.01], *Z* = −2.76, p > 0.01 (Table 5). Stress was also found to be significantly inversely correlated with life satisfaction, with *a* = −0.33, 95% CI [−0.50, −0.17], *Z* = −4.02, *p* < 0.01, and *a* = 0.12, 95% CI [0.06, 0.18], *Z* = 3.79, *p* < 0.01, with an emphasis on planning. Given that full mediation occurs, the direct link between stress and concentration on planning becomes statistically insignificant [*β* = −0.00, CI95% (−0.07, 0.08), *Z* = 0.11, *p* > 0.05; Table 6].

**Table 5:** Mediation estimate for life satisfaction in the relationship between stress and planning refocus.

				95% Confidence interval				
Effect	Label	Estimate	SE	Lower	Upper	*Z*	*p*	% Mediation
Indirect	a × b	−0.04	0.01	−0.07	−0.01	−2.76	0.01	90.54
Direct	c	0.00	0.04	−0.07	0.08	0.11	0.91	9.46
Total	c + a × b	−0.04	0.04	−0.11	0.04	−0.95	0.34	100.00

**Table 6:** Path analysis for life satisfaction in the relationship between stress and planning refocus.

					95% Confidence interval			
		Label	Estimate	SE	Lower	Upper	*Z*	*p*
Stress	→ Life Satisfaction	a	−0.33	0.08	−0.50	−0.17	−4.02	< 0.001
Life satisfaction	→ Refocus on planning	b	0.12	0.03	0.06	0.18	3.79	< 0.001
Stress	→ Refocus on planning	c	0.00	0.04	−0.07	0.08	0.11	0.912

Regression analysis results showed that positive refocusing is positively associated with life satisfaction among youth. Thus, positive refocusing explains 5% of the level of life satisfaction, *R*^2^ = 0.05, *F* (1, 188) = 9.95, *p* < 0.01, *β* = 0.22, *p* < 0.01, the confidence interval being CI95% [0.14, 0.60] (Table 7).

**Table 7:** Regression coefficient for positive refocusing as a predictor of life satisfaction.

Model	*R* ^2^	*B*	ES B	*β*	*t*	*p*
1	0.05	0.37	0.12	0.22	3.16	0.00

Gender and place of origin were not controlled as variables in the study due to the predominance of females in the sample and the majority of participants coming from urban areas. The lack of balance between the two groups could have potentially influenced the results.

## 4 Discussion

In the realm of research concerning stressful life events, the primary emphasis centers on understanding the circumstances surrounding these events, the factors that triggered them, and the resources individuals draw upon to adapt when confronted with such events. Equally crucial is examining how each person subjectively interprets and responds to the events they have experienced (Schwarzer and Luszczynska, 2012).

The study aimed to explore the relationship between critical life events, psycho-emotional health, and life satisfaction among young adults. The results revealed several significant findings that shed light on the complex interplay between these variables. The study confirmed that critical life events have a significant impact on the psycho-emotional health of young individuals, specifically, the absence of critical life events was associated with lower levels of stress, anxiety, and depression, all of which are indicative of better psycho-emotional health. These findings align with previous research that has shown how life events, especially those perceived as highly stressful, can lead to adverse psychological outcomes (Dohrenwend, 2006; Lucas, 2007; Monroe et al., 2007). Individuals who experienced fewer critical life events tended to report better psychological well-being, underlining the importance of preventing or mitigating such events to preserve psycho-emotional health. In support of the above statements, we highlight the findings of several studies in the specialized literature that obtained similar results. For example, the study by Zhang et al. (2020) state that certain events can disrupt a person’s normal functioning balance, especially if they occur at an unexpected moment, which has adverse consequences on the psycho-emotional health of individuals, occurring as an avalanche of negative psychological reactions. This stage was defined as “a brief personal psychological disturbance, precipitated by a danger that produced emotional disturbances and disturbances in the daily routine” (Feinstein, 2021) of the individual, being felt all the more intense as the level of resilience is low at the time of critical events. It was found that a higher tendency toward catastrophizing was associated with lower life satisfaction, while a greater capacity for reappraisal was linked to higher life satisfaction. These results suggest that individuals’ ability to cope with life’s challenges plays a crucial role in their overall life satisfaction. Catastrophizing, which involves exaggerating the negative aspects of a situation, was found to have a detrimental effect on life satisfaction. Conversely, reappraisal, which involves reevaluating situations in a more positive light, was associated with greater life satisfaction. These findings emphasize the importance of teaching individuals effective coping strategies to enhance their overall well-being.

It was observed that a higher degree of emotional regulation through reappraisal was associated with lower levels of stress, anxiety, and depression, indicating better psycho-emotional health. On the other hand, suppression, which involves inhibiting emotional expression, did not show a significant correlation with psycho-emotional health. This suggests that the ability to reevaluate and manage one’s emotions plays a more prominent role in maintaining good psychological well-being than suppressing emotions. These findings underscore the importance of emotional intelligence and adaptive emotional regulation strategies in promoting mental health.

Life satisfaction partially explains the relationship between stress and the tendency to focus on planning. Stress was found to have an inverse relationship with life satisfaction, with higher stress levels associated with lower life satisfaction. Furthermore, positive refocusing was positively associated with life satisfaction, indicating that individuals who engage in positive refocusing tend to report higher life satisfaction. These results highlight the intricate relationship between stress, coping mechanisms, and life satisfaction, emphasizing the need to address stress effectively to enhance overall well-being.

We believe that the subject of this paper is topical, more so since in recent years the specialized literature has mainly focused on the role of critical life events in the emergence of clinical symptoms and how they directly impact life satisfaction. At the same time, the subject of this study is of interest all the more because, starting from the data obtained, the necessary steps can be taken to design programs aimed at increasing the level of resilience in the event of facing critical personal life events and developing coping mechanisms focused on solving problems rather than catastrophizing.

### 4.1 Limitations and future research directions

One of the limits of the present study is represented by the fact that the instruments applied were of the self-report type, being unmoved by objectivity regarding the manner in which the participants provided answers to the questionnaires within the research. At the same time, although significant effects were found, not all the instruments used in the research were adapted to the Romanian population, in their case the translation into Romanian was carried out, for which we did not have enough time to carry out pilot studies.

A second limitation is related to the study sample that did not include a large number of male participants. As a result, generalization of the results should be done with caution.

Also, another limitation of the study regarding the sample is represented by the fact that most of the participants come from the southern region of the country, a fact for which the generalization of the results must be done with caution at the level of other regions.

In future research, we will consider the use of other ways of evaluating participants’ attitudes and opinions regarding the study variables compared to the self-report instruments applied in the current study. At the same time, we propose that in the future we attract in our studies a larger number of men and also a larger number of participants from different regions of the country so that the generalization of the results is as representative as possible for the investigated issue.

Also, carrying out a longitudinal study on the issue of the impact that critical life events from childhood reflect on the subsequent psycho-emotional development of youths can constitute a future direction of research. Moreover, the development of a psychoeducational guide on training the personal ability to distinguish between the factors that trigger and maintain the state of psychological ill health and the protective factors related to a balanced psycho-emotional health is necessary. It can be an informative base for psychosocial specialists, giving youths in need a broad perspective of how exposure to critical life events at any age can lead to health repercussions and the development of maladaptive coping mechanisms.

Lastly, future studies will consider the use of instruments translated and adapted to the Romanian population to capture numerous aspects of the studied constructs.

### 4.2 Conclusion

It is desirable that youth who are currently faced with poor psycho-emotional health, a low level of life satisfaction or with difficulties adapting to their own lives due to the critical events they have faced recently, are oriented towards therapeutic approaches or psychological counseling. These interventions are intended to restore the psycho-emotional balance and provide direction in terms of shaping a therapeutic approach through which people in need discover coping mechanisms focused on solving problems, but also on rationalizing events with a strong emotional impact in order to restore mental balance.

The results of this study add to the current findings regarding the practical implications that critical life events or catastrophizing coping mechanisms impact psycho-emotional health among youth. At the same time, positive refocusing as a coping mechanism is a significant positive predictor of life satisfaction, which can represent a key point in terms of confronting critical life events and restoring psycho-emotional balance.

In this sense, the objective of this research has been achieved, the present study representing a starting point in understanding the manner in which critical life events represent a predisposing factor for the destabilization of mental and emotional health and how specialists in the field of clinical psychology can contribute to restore the psycho-emotional balance among youths.

### 4.3 Practical applicability of research results

The study of the impact that critical life events have on psycho-emotional health is a subject of great importance, because exposure to this type of events that are associated with a significant level of stress can lead over time to the psychological phenomenon called “learned helplessness,” which states that a person is conditioned to believe that they cannot do anything to change the circumstances of their situation (Wu et al., 2013).

Based on these findings, what is currently desired in the field of mental health and clinical psychology is the development of support programs for people who have faced stressful and painful life events such as the loss of a partner, job (Wójcik et al., 2019; APA Dictionary of Psychology, 2022a) or other types of critical events. The focus in these programs is on improving self-esteem, informing people about the negative effects of critical life events or developing coping strategies and emotional regulation. Specialist studies highlight the fact that participation in such programs to raise awareness of the negative effects associated with critical events leads to a decrease in externalizing problems and an early reduction in symptoms associated with mental health disorders or risky behaviors (Obeid et al., 2021; Hokit, 2022).

Therefore, the results of the present study can represent a starting point regarding the development of programs addressed to youths and other people in need, centered on psychoeducation regarding the importance of psycho-emotional health and the way in which it can be diminished following the confrontation with critical life events.

## Data availability statement

The raw data supporting the conclusions of this article will be made available by the authors, without undue reservation.

## Ethics statement

The data contained in this study is part of a dissertation thesis, which has undergone a careful analysis, meeting the necessary ethical criteria. For that reason, ethical review and approval was not required for the study of human participants in accordance with the local legislation and institutional requirements. Written informed consent from the patients/ participants OR patients/participants legal guardian/next of kin was not required to participate in this study in accordance with the national legislation and the institutional requirements.

## Author contributions

GM: Conceptualization, Data curation, Formal analysis, Investigation, Methodology, Project administration, Software, Supervision, Validation, Visualization, Writing – original draft, Writing – review & editing. LM: Conceptualization, Formal analysis, Investigation, Methodology, Software, Validation, Visualization, Writing – original draft, Writing – review & editing, Project administration. C-I-MV: Conceptualization, Data curation, Formal analysis, Investigation, Methodology, Software, Writing – original draft, Writing – review & editing, Validation, Visualization.
